# Evaluation of T-cell aging-related immune phenotypes in the context of biological aging and multimorbidity in the Health and Retirement Study

**DOI:** 10.1186/s12979-022-00290-z

**Published:** 2022-07-20

**Authors:** Ramya Ramasubramanian, Helen C. S. Meier, Sithara Vivek, Eric Klopack, Eileen M. Crimmins, Jessica Faul, Janko Nikolich-Žugich, Bharat Thyagarajan

**Affiliations:** 1 Division of Epidemiology and Community Health, School of Public Health, University of Minnesota, Minneapolis, MN USA; 2 Institute for Social Research, Survey Research Center, University of Michigan, Ann Arbor, MI USA; 3 Department of Laboratory Medicine and Pathology, University of Minnesota, Minneapolis, MN USA; 4 Leonard Davis School of Gerontology, University of Southern California, Los Angeles, CA USA; 5 Department of Immunobiology and the University of Arizona Center On Aging, University of Arizona College of Medicine-Tucson, Tucson, AZ USA

**Keywords:** Immune aging, Adaptive immunity, Biological aging, Multimorbidity, Health and Retirement Study

## Abstract

**Background:**

Cellular changes in adaptive immune system accompany the process of aging and contribute to an aging-related immune phenotype (ARIP) characterized by decrease in naïve T-cells (T_N_) and increase in memory T-cells (T_M_). A population-representative marker of ARIP and its associations with biological aging and age-related chronic conditions have not been studied previously.

**Methods:**

We developed two ARIP indicators based on well understood age-related changes in T cell distribution: T_N_/(T_CM_ (Central Memory) + T_EM_ (Effector Memory) + T_EFF_ (Effector)) (referred as T_N_/T_M_) in CD4 + and CD8 + T-cells. We compared them with existing ARIP measures including CD4/CD8 ratio and CD8 + TN cells by evaluating associations with chronological age and the Klemera Doubal measure of biological age (measured in years) using linear regression, multimorbidity using multinomial logistic regression and two-year mortality using logistic regression.

**Results:**

CD8 + T_N_ and CD8 + T_N_/T_M_ had the strongest inverse association with chronological age (beta estimates: -3.41 and -3.61 respectively; *p*-value < 0.0001) after adjustment for sex, race/ethnicity and CMV status. CD4 + T_N_/T_M_ and CD4 + T_N_ had the strongest inverse association with biological age (β = -0.23; *p* = 0.003 and β = -0.24; *p* = 0.004 respectively) after adjustment for age, sex, race/ethnicity and CMV serostatus. CD4/CD8 ratio was not associated with chronological age or biological age. CD4 + T_N_/T_M_ and CD4 + T_N_ was inversely associated with multimorbidity. For CD4 + T_N_/T_M_, people with 2 chronic conditions had an odds ratio of for 0.74 (95%CI: 0.63–0.86 *p* = 0.0003) compared to those without any chronic conditions while those with 3 chronic conditions had an odds ratio of 0.75 (95% CI: 0.63–0.90; *p* = 0.003) after adjustment for age, sex, race/ethnicity, CMV serostatus, smoking, and BMI. The results for the CD4 + T_N_ subset were very similar to the associations seen with the CD4 + T_N_/T_M_. CD4 + T_N_/T_M_ and CD4 + T_N_ were both associated with two-year mortality (OR = 0.80 (95% CI: 0.67–0.95; *p* = 0.01) and 0.81 (0.70–0.94; *p* = 0.01), respectively).

**Conclusion:**

CD4 + T_N_/T_M_ and CD4 + T_N_ had a stronger association with biological age, age-related morbidity and mortality compared to other ARIP measures. Future longitudinal studies are needed to evaluate the utility of the CD4 + subsets in predicting the risk of aging-related outcomes.

**Supplementary Information:**

The online version contains supplementary material available at 10.1186/s12979-022-00290-z.

## Introduction

Aging is a complex process accompanied by changes in the immune system which might result in the acceleration of biological decline and incidence of chronic diseases [1]. Immune system aging reduces the ability of the immune system to mount effective immune responses against new infections, vaccinations, and antigens [2, 3]. Previous studies have consistently reported that aging-related changes in T cells [4–6] contribute to an aging-related immune phenotype (ARIP) that is characterized by a reduced T-cell repertoire, reduction of naïve T cells (T_N_), and accumulation of memory (T_M_) and effector T cells [7, 8]. Though the determinants of ARIP remain incompletely understood, age, sex, and exposure to cytomegalovirus (CMV) are major determinants of T cell subsets [9–12]. In our previous study [13], we evaluated the associations of individual T-cell subsets measured in the Health and Retirement Study with chronological age. The study showed that CD4 + and CD8 + T_N_ decreased with age and CD4 + T_N_ cells were substantially higher among CMV seronegative individuals and women. We observed that CD8 + T_EFF_, CD4 + T_EFF,_ and CD4 + T_EM_ cells were strongly influenced by CMV seropositivity, and CD8 + T_EM_ increased with age. CD8 + and CD4 + T_CM_ decreased with age although CD4 + T_CM_ was higher among women compared to men and CD8 + T_CM_ was higher among men. These results show that the inter-individual variability in T cell subsets is determined by several factors such as age, sex and CMV serostatus and individual T cell subsets are differentially influenced by the various factors. Optimal T-cell immunity involves harmonized action between multiple cell populations and a population level metric combining the cell populations will be biologically meaningful in understanding the aging immune system. In this regard, CD4/CD8 ratio is a well-established ARIP measure where the prevalence of an inverted CD4/CD8 ratio increases with age [14, 15]. However, we have shown previously that the prevalence of the inverted CD4/CD8 ratio increases with age only among CMV seropositive individuals and not among CMV seronegative individuals suggesting that this ratio is not a universal marker of ARIP as previously thought [13]. Thus, other measures of ARIP, beyond the inverted CD4/CD8 ratio, that are independent of CMV serostatus need to be identified. To this end, one study identified a cellular composite measure (IMM-AGE) that was associated with mortality, and another study developed an inflammatory aging clock (iAGE) utilizing soluble systemic chronic inflammation markers or cytokines which was associated with multimorbidity [16, 17]. However, the interpretation of cytokine levels is complicated by the fact that that circulating cytokine levels are influenced by secretion from multiple cell types and the IMM-Age measure that utilized a combination of flow cytometry and mass cytometry estimated differences in both innate and adaptive immune systems. However, since age-related and CMV-related changes are seen predominantly in the T-cell subsets, a composite measure focused on T cells and relatively easy to implement in large epidemiological studies is needed.

To address this gap, we used T-cell immunophenotyping data that measured 11 T cell subsets in a large, population-based sample and used well understood aging-related changes in T cell distribution to develop aging-related immune phenotype (ARIP) indicators that would be robust across sexes, race/ethnic groups and CMV serostatus. We developed ARIP measures based on a priori knowledge of (i) the role of chronological age in the immune system and (ii) the role of underlying biological mechanisms in the immune system such as CMV which increases susceptibility to age-related chronic conditions [18–20].We also evaluated the correlation between individual T-cell subsets and association of individual T-cell subsets with chronological age, biological age and multimorbidity to develop composite ARIP measures. We created two candidate measures: T_N_/ (T_CM_ + T_EM_ + T_EFF_) (referred as T_N_/ T_M_) in CD4 + and CD8 + T cells and benchmarked them against CD4/CD8 ratio, CD8 + T_N_ and CD4 + T_N_ by evaluating associations of all these measures with chronological age, biological age, multimorbidity (that includes several aging-related diseases such as diabetes, cancer, lung disease, stroke, and heart disease), and mortality.

## Methods

### Study Population

The Health and Retirement Study (HRS), supported by National Institute of Aging (NIA), is a nationally representative longitudinal survey of adults in the United States over the age of 50. The study began in 1992 with participants 51–61 years of age and now uses a steady-state design to replenish the sample every 6 years with younger cohorts [21, 22] with surveys/interviews being conducted biennially. The HRS sample employs a multi-stage area probability design with oversampling of African-American and Hispanic households at about twice the rate of Whites [21]. Whole blood was collected from HRS participants in the 2016 wave as part of the Venous Blood Study (VBS) and immunophenotyping was performed on samples from 9932 participants. After removing participants with missing age, sex, race/ethnicity, CMV seroprevalence, Venous Blood Study survey weights, and T cell data, 8603 participants were included in the study. After weighting the sample using Venous Blood Study survey weights, it is representative of a U.S older national population.

### Immunophenotyping

Peripheral Blood Mononuclear cells (PBMCs) were isolated from whole blood and used for the measurement of immune cells [23]. All flow cytometry measurements were performed on an LSRII flow cytometer or a Fortessa X20 instrument (BD Biosciences, San Diego, CA). Immunophenotyping data was analyzed using OpenCyto and FlowAnnotator as described previously [24]. T-cells and ten T-cell subsets were evaluated in HRS. Panel 1 consisted of antibody cocktails targeting T cells and T cell subsets (Supplementary Table 1). T cell subsets were represented as percentages of their parent population. The detailed methods and cell subset definitions have been mentioned previously [13].

### Ascertainment of multimorbidity and mortality

Prevalence of type II diabetes, stroke, lung disease, heart disease, and cancer were used to generate a 2016 multimorbidity outcome. Type II diabetes was defined as fasting glucose value >  = 126 mg/dL or use of diabetes medications or insulin. Stroke, lung disease, heart disease and cancer were obtained from self-report. Multimorbidity was defined as a categorical variable with four categories (no prevalent chronic conditions (reference), a single chronic condition, two chronic conditions, and three or more chronic conditions). Incident multimorbidity in 2018 and 2020 was ascertained by defining a baseline study population with participants who had a multimorbidity score of 0 or 1 in 2016. Among the participants in this baseline population, incident multimorbidity was defined as a multimorbidity score of 2 or higher in 2018 and 2020 using self-report of Type II diabetes, stroke, lung disease, heart disease, and cancer. Mortality assessed at 2018 was used for this analysis. This measurement is based on HRS tracking efforts when there is no interview in the current wave, or an exit interview was not obtained in the prior wave. The reported vital status is based on reports from someone capable of reporting a death.

### Ascertainment of biological age and self-rated health

Biological age was developed using the Klemera Doubal [25] method as a combination of biomarkers which represents the decline in aging-related physiological functioning and susceptibility to disease in old age. It was previously validated in the NHANES cohort as a more reliable predictor of mortality than chronological age and other biological age algorithms [26]. The biomarkers used for calculating the biological age were: Cardiovascular function (Systolic Blood pressure), Metabolic markers (Total Cholesterol and Fasting Glucose), Inflammation (CMV and C-Reactive Protein), Kidney function (Serum Creatinine and Blood Urea Nitrogen), Liver function (Alkaline phosphatase and Albumin), Lung function (Peak flow). Biological age was calculated using the R package BioAge and the in-built function kdm_calc [27]. Self-rated health was measured in the Health and Retirement Study by asking the question “Would you say your health is excellent, very good, good, fair, or poor?” We combined the “excellent”, “very good” and “good” categories to represent a good score for health and the “fair” and “poor” categories to represent a bad score for health.

### Ascertainment of participant characteristics and biological age

Chronological age (in years), sex (female/male), race/ethnicity (Hispanic, Non-Hispanic Whites, Non-Hispanic Black, and Non-Hispanic Other) were obtained from the HRS demographic data [22]. Smoking was self-reported and categorized as never smokers, former smokers, and current smokers. Height was measured in inches and weight was measured in pounds. Height and weight for all the participants were obtained for half the sample in 2014 and the other half in 2016. BMI was calculated by using the formula (Weight in lbs/ (Height in inches)^2^) *703. CMV seroprevalence was measured using the ratio of total IgG to anti-CMV IgG in serum using the Roche e411 immunoassay analyzer (Roche Diagnostics Corporation). The results are reported as non-reactive (< 0.5 COI), borderline (0.5 to < 1.0 COI) or reactive >  = 1.0 COI) where COI is cut-off interval [28]. This was used as a binary variable by combining the borderline and negative groups.

### Statistical analysis

We evaluated two candidate ARIP measures (T_N_/T_M_ CD4 + and CD8 + T cells) to compare with existing CD4/CD8 ratio and the individual cell type CD8 + TN as we have shown previously that it had a strong inverse association with chronological age [13]. We studied associations of the candidate ARIP and existing ARIP measures with chronological age, biological age, and multimorbidity. CD4 + T_N_/T_M_, CD8 + T_N_/T_M_ and CD4/CD8 ratio were log-transformed due to skewed distributions.

Pearson correlation coefficients were calculated between individual T-cell subsets. To study the association of ARIP measures with chronological age, and biological age, survey linear regression models were used after adjustment for chronological age, sex, race/ethnicity, and CMV status. A biological age acceleration measure was also calculated by subtracting chronological age from biological age. The participants were categorized into “higher” category if the age acceleration measure was higher than 0, and the participants were grouped into “lower” category if the age acceleration measure was lower than 0. Using the “lower” group as a reference, logistic regression analysis was performed between ARIP measures and biological age after adjustment for age, sex, race/ethnicity and CMV status. To study the association of ARIP measures with prevalent multimorbidity in 2016, multinomial survey logistic regression models were used. We studied the association between ARIP measures and the individual components of multimorbidity (type II diabetes, hypertension, lung disease, heart disease, and stroke) by using a series of survey logistic regression models after adjustment for age, sex, race/ethnicity, CMV status, smoking status, and BMI. We used survey cox regression models to study the association of ARIP measures in incident multimorbidity in 2018 and 2020. We used survey logistic regression models to study the association of the ARIP measures with self-rated health and mortality in 2018. The survey models were adjusted for survey design parameters including strata and cluster details for sampling error and participant sample weights from the Venous Blood Study to account for sample design. In sensitivity analyses, three additional characterizations of ARIP were explored and results are presented in the supplementary material: (i) CD4 + T_N_/CD8 + T_N_; (ii) CD4 + T_N_ + T_CM_; (iii) CD8 T_N_ + T_CM_.

We used SAS version 9.4 (SAS Institute, Inc., Cary, NC) and R Statistical Analysis software version 4.0.0 for all the analyses.

## Results

### Participant characteristics

Among the 8603 participants included, the average age of participants was 68.65 years. Fifty-four percent of the participants were women; 10.02% of participants were Non-Hispanic Black and 9.01% were Hispanic; 11.87% of the participants were current smokers and the average BMI was 29.69 kg/m^2^. Sixty-four percent of the participants were seropositive for cytomegalovirus. In 2016, 48.13% of participants were without any chronic conditions, 32.82% participants with one chronic condition, 14.06% participants with two chronic conditions, and 4.98% participants with three or more chronic conditions. Heart disease was the most prevalent (24.83%) among the diseases used to define multimorbidity (Table 1). Six percent of the participants had incident multimorbidity in 2018 and 2020, and four percent of the participants were not alive in 2018 (Table 1). As expected, CD4 + T_N_ was negatively correlated with CD4 + T_CM_, CD4 + T_EFF_ and CD4 + T_EM_ (*R* = -0.72, -0.3 and -0.22, respectively) (Supplementary Fig. 1). CD8 + T_N_ was negatively correlated with CD8 + T_EFF_ and CD8 + T_EM_ (*R* = -0.67 and -0.26, respectively) (Supplementary Fig. 1). Based on the negative correlation measures of T_N_ with T_CM_, T_EFF_, and T_EM,_ the composite ARIP measures CD4 + and CD8 + T_N_ /(T_CM_ + T_EM_ + T_EFF_) (referred as T_N_/ T_M_) were developed.

**Table 1:** Descriptive statistics of Health and Retirement Study participant characteristics measured in 2016 among 8603 participants with immunophenotyping data

Characteristics	Mean ± SE/ Frequency (%)
**Demographics**	
Age (years) (Mean ± SE)	68.65 ± 0.26
Sex – Female (%)	54.12%
Race/Ethnicity	
Hispanic (%)	9.01%
Non-Hispanic Black (%)	10.02%
Non-Hispanic White (%)	77.55%
**Lifestyle characteristics**	
Smoking Status	
Never smokers (%)	44.29%
Former smokers (%)	43.84%
Current smokers (%)	11.87%
BMI (kg/m^2^) (Mean ± SE)	29.69 ± 0.10
Cytomegalovirus seroprevalence – Reactive (%)	64.54%
Biological Age (years) (Mean ± SE)	67.66 ± 0.24
**Prevalence of disease conditions in 2016**	
Multimorbidity score	
0	48.13%
1	32.82%
2	14.06%
3 and above	4.98%
Type II diabetes (%)	17.55%
Heart Disease (%)	24.83%
Stroke (%)	7.04%
Lung Disease (%)	11.32%
Cancer (%)	16.19%
Percentage of participants with bad score for self-rated health in 2016	26.61%
Mortality in 2018	4.17%
Number of people with incident multimorbidity in 2018 and 2020	6.01%
**Age-related immune phenotype measures**	
CD4 + /CD8 + (Mean ± SD) **(log-transformed)**	1.09 ± 0.70
T_N_/T_M_ CD4 + (Mean ± SD) **(log-transformed)**	-0.03 ± 0.77
T_N_/T_M_ CD8 + (Mean ± SD) **(log-transformed)**	-1.04 ± 1.14
CD8 + T_N_	0.22 ± 0.16
CD4 + T_N_	0.43 ± 0.18

### Association of ARIP measures with chronological age

**Fig. 1:**
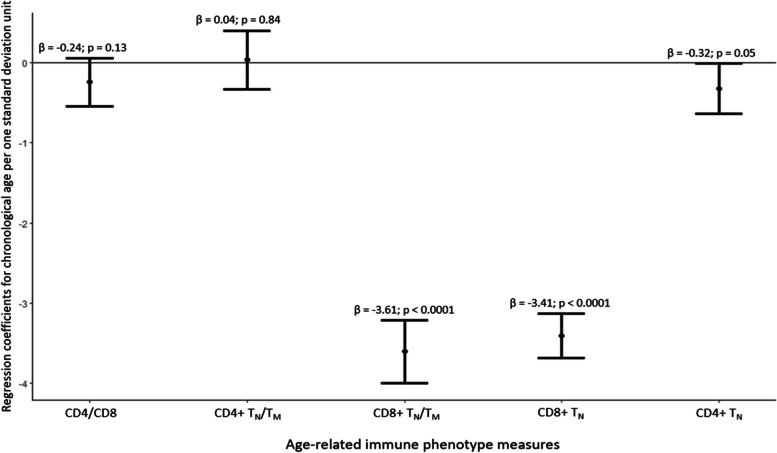
Association of ARIP measures with chronological age. Age is used as the dependent variable in survey linear regression models. The beta estimates are estimated per one standard deviation unit of the ARIP measures. The models are adjusted for sex, race/ethnicity, and CMV status. The solid black line along 0 indicates no association

Overall, the CD8 + subsets were more strongly associated with chronological age than the CD4 + subsets. Among the individual cell subsets, CD4 + and CD8 + T_N_ were negatively associated with chronological age (β = -0.32; *p* = 0.05 and β = -3.41; *p* < 0.0001). CD4 + and CD8 + T_EFF_ (β = 0.66; *p* < 0.0001 and β = 2.58; *p* < 0.0001), CD4 + and CD8 + T_EM_ (β = 1.81; *p* < 0.0001 and β = 1.7; *p* < 0.0001) and CD8 + T_CM_ (β = 0.39; *p* = 0.04) were positively associated with chronological age (Supplementary Fig. 2). Among the ARIP measures, univariate analysis showed that CD4/CD8, CD4 + T_N_/T_M,_ and CD4 + T_N_ had the weakest association with chronological age whereas CD8 + T_N_/T_M_ and CD8 + T_N_ had the strongest association with chronological age (Supplementary Fig. 3). A one standard deviation (SD) increase in CD8 + T_N_/T_M_ (*p* < 0.001) was associated with a 3.61 years lower chronological age (Fig. 1) after adjusting for sex, race/ethnicity, and CMV status. CD4/CD8 and CD4 + T_N_ /T_M_ were not associated with chronological age (*p* = 0.13 and *p* = 0.84, respectively). The other ARIP measures also had an inverse association with chronological age (Supplementary table 2).

### Association of ARIP measures with biological age

**Fig. 2:**
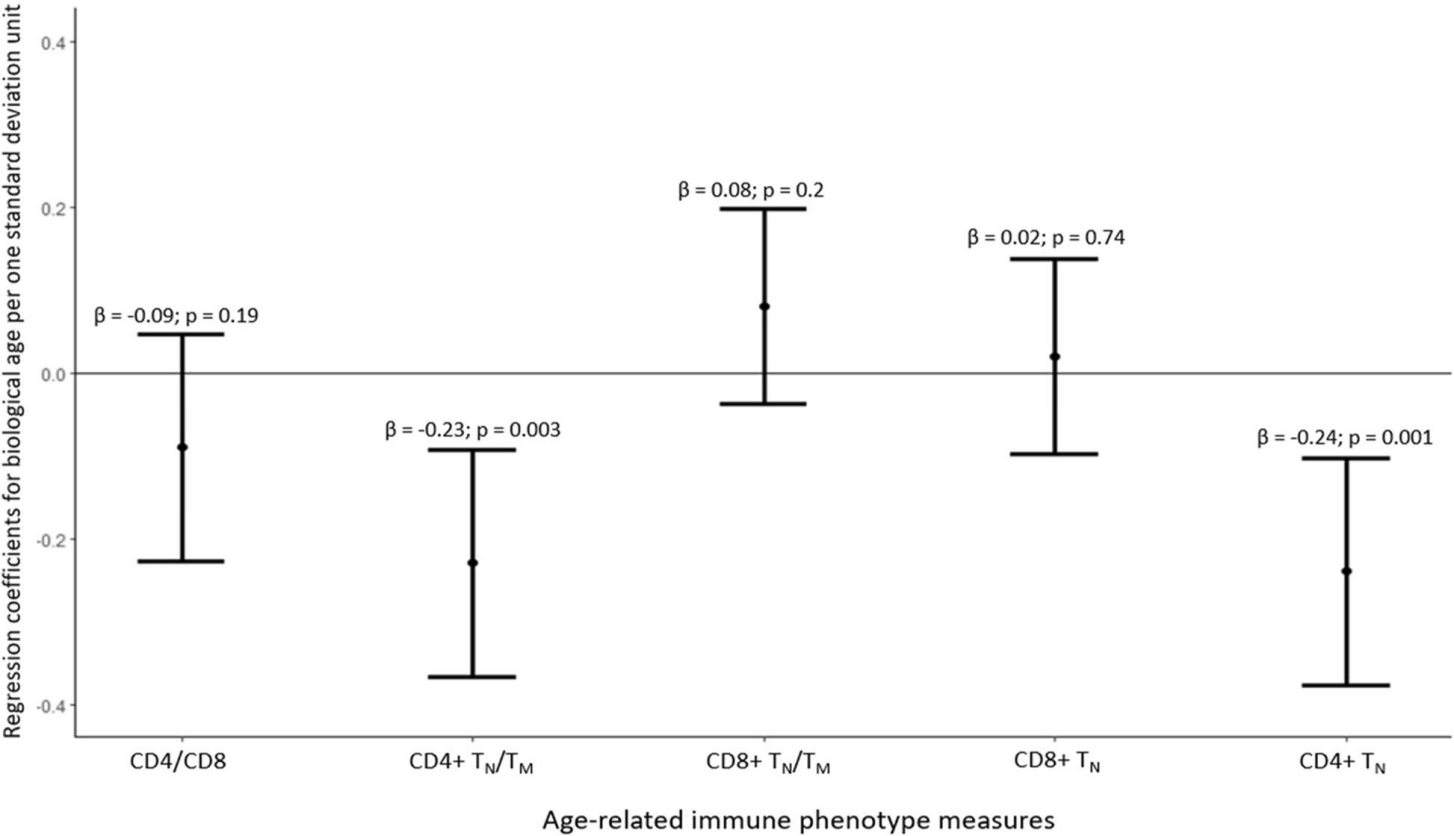
Association of ARIP measures with biological age. Biological age is used as the dependent variable in survey linear regression models. The beta estimates are estimated per one standard deviation unit of the ARIP measures. The models are adjusted for chronological age, sex, race/ethnicity, and CMV status. The solid black line along 0 indicates no association

Among the individual T-cell subsets, CD4 + T_N_ was negatively associated with biological age (0.24 years decrease in biological age for one standard deviation (SD) increase in CD4 + T_N_ (*p* = 0.001) (Fig. 2), one SD increase in CD4 + T_CM_ and CD4 + T_EFF_ was associated with 0.18 years (*p* = 0.01) and 0.13 years (*p* = 0.02) higher biological age respectively (Supplementary Fig. 4). Among the CD8 + T cell subsets, only the CD8 + T_CM_ subset was positively associated with biological age (0.12 years higher biological age for one SD increase in CD8 + T_CM_ (*p* = 0.03)) (Supplementary Fig. 4). Univariate analysis showed that CD4/CD8, CD4 + T_N_/T_M,_ and CD4 + T_N_ had the weakest association with biological age whereas CD8 + T_N_/T_M_ and CD8 + T_N_ had the strongest association with biological age (Supplementary Fig. 5). However, after adjustment for covariates, CD4 + T_N_/T_M_ had a strong negative association with biological age with 0.23 years lower biological age for one standard deviation increase in CD4 + T_N_/T_M_ (*p*-value = 0.003) (Fig. 2). CD4/CD8 ratio, CD8 + T_N_/T_M,_ and CD8 + T_N_ were not associated with biological age. CD4 + T_N_/T_M_,CD4 + T_N_ and CD4 + T_CM_ remained associated with biological age acceleration (older category). One SD increase in CD4 + T_N_/T_M_ was associated with 8% lower odds of having higher biological age as compared to chronological age (OR: 0.92 (95% CI: 0.84 – 1.00); *p*-value = 0.05), one SD increase in CD4 + T_N_ was associated with 11% lower odds of having higher biological age (OR: 0.89 (95% CI: 0.83 – 0.95; *p* = 0.001) and one SD increase in CD4 + T_CM_ was associated with 9% higher odds of having higher biological age (OR: 1.09 (95% CI: 1.02–1.17; *p* = 0.01). CD8 + T_N_/T_M_ was not associated with biological age acceleration (OR: 1.04; (95% CI: 0.98 – 1.11; *p*-value = 0.19) (Supplementary table 3). Additional adjustment for CRP did not substantially change the associations between ARIP measures and biological age (data not shown).

### Association of ARIP measures with multimorbidity

**Fig. 3:**
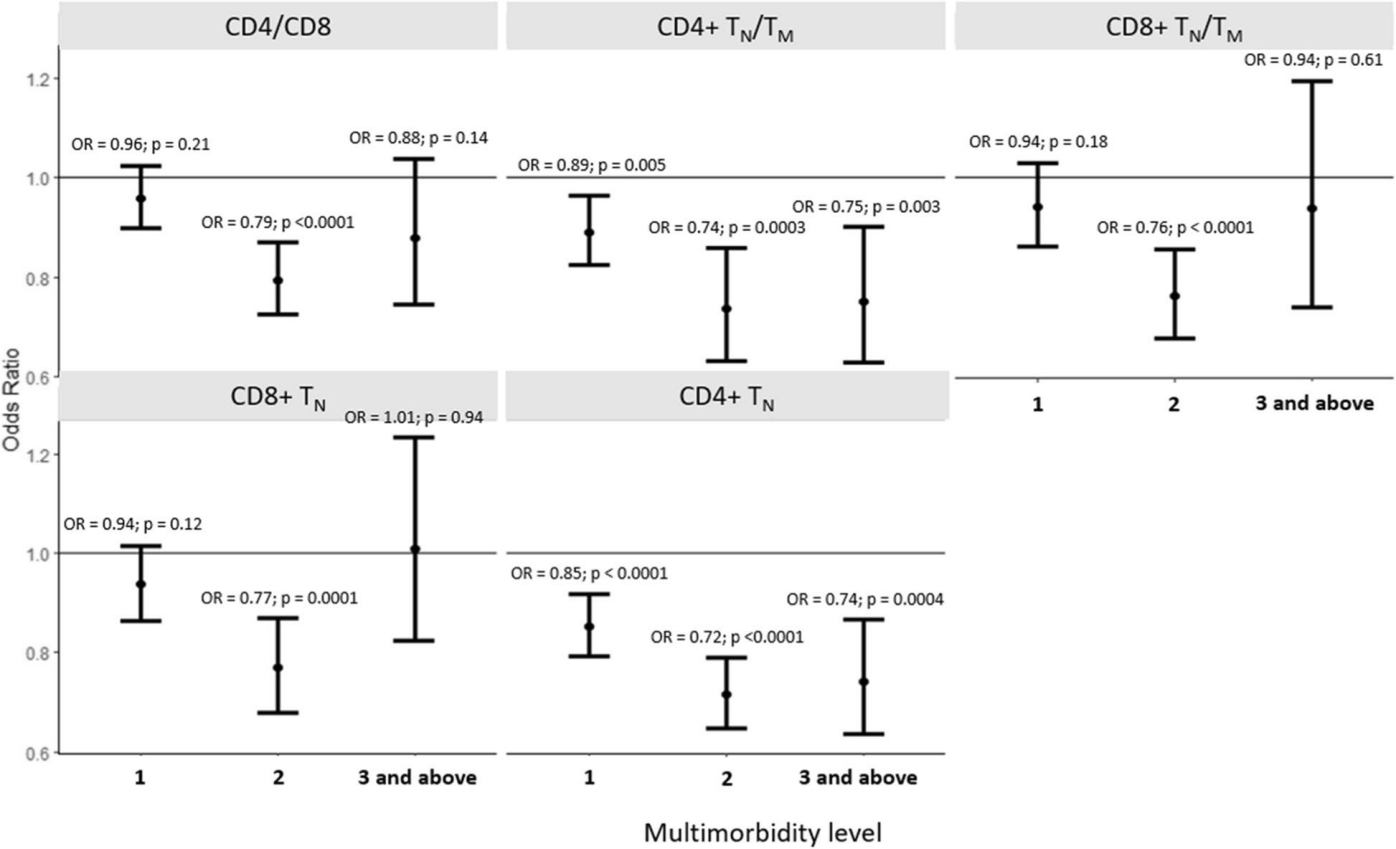
Odds ratios and 95% CI of association of multimorbidity levels with ARIP markers per one SD unit increase in ARIP marker. Adjusted for age, sex, race/ethnicity, CMV status, smoking status, and BMI. The solid black line along OR of 1 indicates no association

**Fig. 4:**
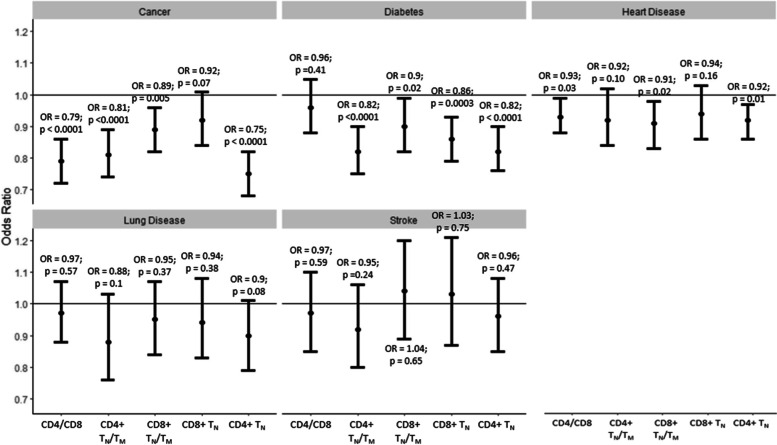
Odds ratios and 95% CI of association of individual components of multimorbidity ARIP markers per one SD unit increase in ARIP marker. Adjusted for age, sex, race/ethnicity, CMV status, smoking status, and BMI. The solid black line along OR of 1 indicates no association

CD4 + T_N_ had an odds ratio of 0.85 (95% CI: 0.79–0.92; *p* < 0.0001) when comparing those with a single chronic condition (multimorbidity score = 1) vs. those without any chronic condition (multimorbidity score = 0), while the corresponding odds ratio for those with two and 3 + chronic conditions was 0.72 (95% CI: 0.65 – 0.79; *p* < 0.0001) and 0.74 (95% CI: 0.64–0.87; *p* = 0.0004) respectively (Fig. 3). Among the other individual T-cell subsets, CD4 + T_CM_ was consistently positively associated across different categories of multimorbidity whereas the other T-cell subsets did not have a consistent association across multimorbidity categories (Supplementary Fig. 6). CD4 + T_N_/T_M_ had an odds ratio of 0.89 (95% CI: 0.82–0.96; *p* = 0.005) when comparing those with a single chronic condition vs. those without any chronic condition, while the corresponding odds ratio was 0.74 (95% CI: 0.63 – 0.86; *p* = 0.0003) and 0.75 (95% CI: 0.63–0.90; *p* = 0.003) for those with two and 3 + chronic conditions, respectively (Fig. 3). CD4/CD8, CD8 + T_N_/T_M_ and CD8 + T_N_ were not consistently associated across the different categories of multimorbidity (Fig. 3). Further stratifying the highest level of the multimorbidity score into 3 and 4 + did not change the observed associations (data not shown). Among the chronic diseases included in the multimorbidity score, ARIP measures had the strongest association with prevalent cancer after multivariate adjustment. Lung disease, diabetes, stroke, and heart disease were not individually associated with the ARIP measures after multivariate adjustment (Fig. 4, Supplementary Table 4). CD4 + T_CM_ was associated with incident multimorbidity over four years follow-up (OR: 1.09 (95% CI: 1.01 – 1.18; *p*-value = 0.03). CD4 + T_N_/T_M_ and CD4 + T_N_ also had association with incident multimorbidity in the consistent direction (OR: 0.91 (95% CI: 0.82- 1.02); *p*-value = 0.09 and OR = 0.91 (95% CI: 0.82 – 1.01); *p*-value = 0.09) though the associations were not statistically significant. (Supplementary Table 4).

### Association of ARIP measures with self-rated health and two-year mortality

**Fig. 5:**
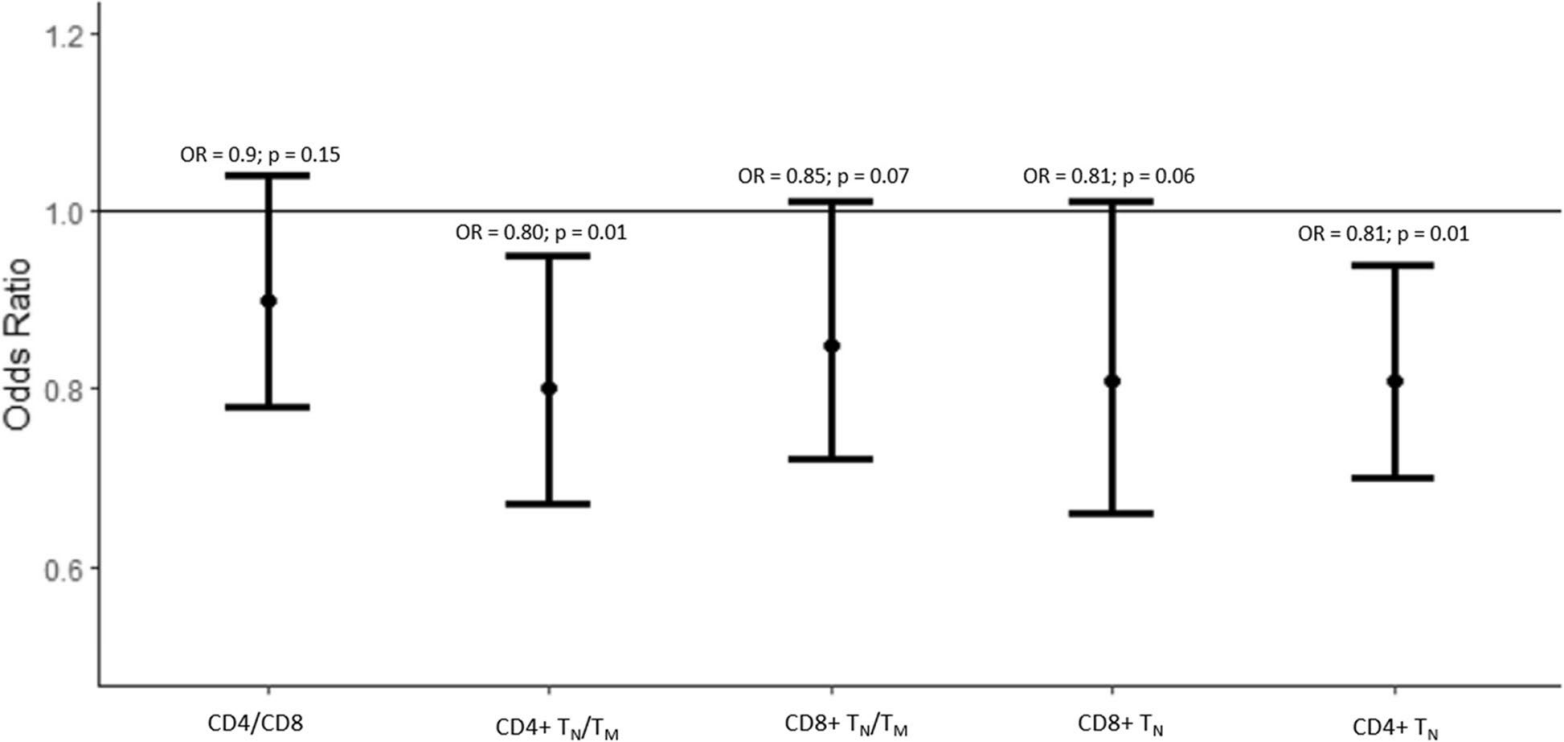
Odds ratios and 95% CI of association of mortality with ARIP markers per one SD unit increase in ARIP marker. Adjusted for age, sex, race/ethnicity, CMV status, smoking status, and BMI. The solid black line along OR of 1 indicates no association

All the ARIP measures were associated with self-reported health (bad score for health vs good score for health) with the strongest association in CD4 + T_N_/T_M_, CD4 + T_N_ and CD4 + T_CM_ with OR of 0.84 (95% CI: 0.78 – 0.91), 0.83 (95% CI: 0.77 – 0.89) and 1.14 (95% CI: 1.04–1.25; *p* = 0.006), respectively (Supplementary table 5). The other individual T cell subsets were not associated with self-rated health (data not shown). CD4 + T_N_/T_M_ and CD4 + T_N_ had the strongest association with mortality with an OR of 0.80 (95% CI: 0.67 – 0.95; *p* = 0.01) and 0.81 (95% CI: 0.70–0.94; *p* = 0.01), respectively. The other ARIP measures were not associated with mortality in 2018 (Fig. 5). Among the individual T-cell subsets, only CD8 + T_EFF_ (OR = 1.27 (95% CI: 1.09–1.49; *p* = 0.003) was associated with mortality in 2018.

## Discussion

This study explored the associations of ARIP measures with chronological age, biological age, and multimorbidity outcomes. CD4 + T_N_/T_M_ and CD4 + T_N_ had the strongest association with biological age and mortality and was inversely associated with increasing levels of multimorbidity. Though CD8 + T_N_/T_M_ was not associated with biological age nor mortality, CD8 + T_N_/T_M_ had associations with individual conditions including heart disease, diabetes and cancer. More examination is needed to better understand the relationship between CD8 + T_N_/T_M_ and the individual chronic conditions. CD4/CD8 ratio was not associated with chronological age or biological age and did not have a consistent association with multimorbidity (Table 2). The associations with increasing levels of multimorbidity, mortality and biological age indicates the CD4 + T_N_/T_M_ and CD4 + T_N_ may be the biomarkers to identify individuals at higher risk for in age acceleration and its associated morbidities and higher mortality.

**Table 2:** Summarizing the associations of the candidate and existing ARIP measures with chronological age, biological age, and multimorbidity

	Chronological Age	Biological Age	Multimorbidity	Mortality
**Existing ARIP measure**				
CD4/CD8	No	No	No (inverse association with increasing levels of multimorbidity)	No
CD8 + T_N_	Yes (inverse)	No	No (inverse association with increasing levels of multimorbidity)	No
CD4 + T_N_	Yes	Yes	Yes (inverse association with increasing levels of multimorbidity)	Yes
CD4 + T_CM_	No	Yes	Yes (positive association with increasing levels of multimorbidity)	No
**Candidate ARIP measure**				
CD8 + T_N_/T_M_	Yes	No	No (inverse association with increasing levels of multimorbidity)	No
CD4 T_N_/T_M_	No	Yes	Yes (inverse association with increasing levels of multimorbidity)	Yes

CD4 + T_N_/T_M_ and CD4 + T_N_ had associations of similar strengths with biological age, multimorbidity and mortality. CD4 + T_N_ and CD4 + T_CM_ were strongly negatively correlated. While CD4 + T_N_ was inversely associated with biological age and multimorbidity, CD4 + T_CM_ was positively associated with biological age and multimorbidity. These observed associations indicate antagonistic roles of CD4 + T_N_ and CD4 + T_CM_ in biological aging and the pathogenesis of age-related chronic conditions. Hence, a representation of ARIP as a combination of CD4 + T_N_/T_M_ may be a better representation of the overall associations between T cell immunity and health outcomes when compared to describing these associations with individual T-cell subsets. However, CD4 + T_N_ also shows similar associations with biological age, multimorbidity and mortality as CD4 + T_N_/T_M._

Among the two new measures developed in this study, CD8 + T_N_/T_M_ was associated with chronological age but not associated with biological age and higher chronic conditions. CD4 + T_N_/T_M_ was not associated with chronological age but had a strong inverse association with biological age and with a higher number of prevalent co-occurring chronic conditions. The findings with CD4 + T_N_/T_M_ are consistent with previous studies where CD4 + T cell subsets were associated with chronic diseases. In the Multi-Ethnic Study of Atherosclerosis (MESA), naïve and memory CD4 + T cells were cross-sectionally associated with type II diabetes and subclinical atherosclerosis [29, 30]. CD4 + T cells produce interferon- γ (IFN- γ) which contributes to inflammation, glucose intolerance, and insulin resistance in diet-induced obesity mice [31, 32]. Another study performed among type II diabetes patients found a significant reduction in the naïve pool in both CD4 + and CD8 + populations and a significant rise in T_EM_ and T_EFF_ populations of the CD4 + subset compared to age‐matched controls [33]. Decreased CD4 + T_N_/T_M_ ratio has also been observed in 76 non-small cell lung cancer (NSCLC) patients compared to 28 age and sex-matched healthy volunteers [34]. CD4 + CD28null cells which have advanced effector functions have been found at an increased frequency in ischemic stroke patients which was also associated with stroke severity [35]. These studies are consistent with our findings that the distribution of naïve and memory CD4 + T cells may be important in determining age-related outcomes. In contrast, the CD8 + T_N_ cells were inversely associated with chronological age after adjustment for sex, race/ethnicity, and CMV serostatus but not associated with biological age and a higher number of chronic conditions. Previous studies have also used CD4/CD8 ratio as a measure of immune aging. An increased prevalence of the inverted CD4/CD8 ratio was associated with short-term mortality in the OCTO immune longitudinal study, possibly due to confounding by CMV serostatus [36]. CD4/CD8 ratio was not associated with biological age and chronological age after adjustment for race/ethnicity, sex, and CMV status and was not consistently associated with a higher prevalence of multiple chronic diseases.

Although increasing chronological age is an important component of senescence, it does not directly measure the accelerated decline in health among individuals. Previous studies have shown that biological age predicts morbidity and mortality independent of chronological age in individuals [26, 37–39]. This is the first study to demonstrate an association between biological age and CD4 + T_N_/T_M_, CD4 + T_N_ and CD4 + T_CM_. In the Rotterdam study, longitudinal associations of biological age with all-cause morbidity, stroke, cancer, and diabetes mellitus, suggests that biological age may predetermine disease occurrence [39]. The strong association between multiple CD4 + subsets including CD4 + T_N_/T_M_ and biological age suggests that CD4 + T_N_/T_M_ might be an important risk factor for future disease occurrence and this needs to be confirmed in future studies.

Heterogeneity in strength of the association between the different components of multimorbidity and the ARIP measures were observed but the direction of the association was consistent among the diseases examined. CD4 + T_N_/T_M_ and CD4 + T_N_ were lower among participants who had one chronic condition (multimorbidity score = 1) compared to those without any chronic conditions (multimorbidity score = 0). Since cancer was the third most frequent chronic condition (after heart disease and diabetes), this may reflect the strong association between cancer and CD4 + T cells. The distribution of chronic conditions when participants had two chronic conditions (multimorbidity score = 2) is similar to the overall prevalence of chronic conditions. Since the CD4 + T_N_/T_M_ and CD4 + T_N_ CD4 subsets were associated with the three most common chronic conditions (heart disease, diabetes and cancer), this could explain the significant association between these subsests and multimorbidity score of two. However, when participants had three or more chronic conditions (multimorbidity score = 3), lung disease had a higher prevalence compared to when participants had two chronic conditions (multimorbidity score = 2). Though none of the ARIP measures were significantly associated with lung disease, CD4 + T_N_/T_M_ and CD4 + T_N_ had the strongest association (OR = 0.88 and 0.90 respectively) with lung disease. This may explain why only CD4 + T_N_/T_M_ and CD4 + T_N_ was associated with a multimorbidity score of 3. Among the disease conditions which constituted multimorbidity, cancer had the strongest association with the ARIP measures. Various theories have been proposed describing the involvement of naïve and memory CD4 + T cells in tumor immunity. The tumor microenvironment conditions promote the differentiation of memory and naïve CD4 + T cells into CD4 + T regulatory cells which suppress antitumor immunity [40–42]. The concept of immunosurveillance in cancer indicates that the immune system can recognize and proactively remove precursors of cancer and naïve T cells may be involved in this process as they perform immunosurveillance roles to undergo proliferation in response to homeostatic signals [43, 44]. In general, T_CM_ cells have been found to produce higher levels of cytokines which have increased efficiency in an antitumor response [45, 46]. We also observed that the ARIP measures were not significantly associated with incident multimorbidity after adjustment for age, sex, race/ethnicity, CMV status, smoking status, and BMI though the direction of association was consistent with what was observed in the cross-sectional analysis. This could be due to the limited number of incident multimorbidity events in the short follow-up period and the association between the ARIP measures and incident multimorbidity needs to be further evaluated in future studies. CD4 + T_N_/T_M_ and CD4 + T_N_ were associated with two-year mortality as well strengthening their role in determining biological aging.

Major strengths of this study include the standardized and rigorous immunophenotyping methods used for measuring the cell populations, representative sample including Hispanic, black, and white individuals, immunophenotyping data available for a large sample size (about 9938 individuals), and concurrent measurement of cytomegalovirus seroprevalence. The novelty of this study is the creation of composite ARIP measures as a marker for an aspect of aging and characterizing the measures using chronological age, biological age, and age-related health outcomes. A limitation of this study is the absence of longitudinal immunophenotype data. Measurement of longitudinal changes in the immune cell subsets would help in studying the change in immune cells with age within the same participant and would help us to better understand the temporal relationship between immune aging and disease. The majority of the multimorbidity measure is based on the self-report of the participant and hence could be influenced by factors such as recall, social desirability, and lack of diagnosis. A previous study found that the sensitivity of self-report of hypertension was 88.9% among blacks, 82.8% for whites, and 84.0% for Hispanic ethnicity, and specificity was 92.8% for the whites and 86% for the blacks [47] informing that the measurements are robust. Another study performed to validate self-reported cancer measures with Medicare claims data had a 73.2% sensitivity and 96.2% specificity [48].

In conclusion, the results suggest that composite measures of age-related immune phenotypes are more meaningful in determining biological aging and multimorbidity. In this study, we showed that CD4 + T_N_/T_M_ and CD4 + T_N_ had strong associations with biological age, multimorbidity and mortality and maybe a more consistent measure of immune aging compared to specific cell types such as CD8 + naïve T cells and the widely used CD4/CD8. CD4 + T_N_/T_M_ and CD4 + T_N_ can be used in future studies with longitudinal measurements of the immune cells to better understand the role of immune aging in age-related morbidity and mortality.

## Supplementary Information

**Additional file 1: Table S1.** T cell subset definitions measured in the Health and Retirement Study. **Figure S1.** Pearson correlation heatmap between individual T-cell subsets measured in the Health and Retirement Study. **Figure S2.** Association of individual T-cell subsets with chronological age. Age is used as the dependent variable in survey linear regression models. The beta estimates are estimated per one standard deviation unit of individual T-cell subsets. The models are adjusted for sex, race/ethnicity, and CMV status. The solid black line along 0 indicates no association. **Figure S3.** Scatterplot of ARIP measures with chronological age with a LOWESS curve. **Figure S4.** Association of individual T-cell subsets with biological age. Biological age is used as the dependent variable in survey linear regression models. The beta estimates are estimated per one standard deviation unit of individual T-cell subsets. The models are adjusted for chronological sex, race/ethnicity, and CMV status. The solid black line along 0 indicates no association. **Figure S5.** Scatterplot of ARIP measures with biological age with a LOWESS curve. **Table S2.** Beta estimates of the association between the additional ARIP measures with chronological age and biological age. **Table S3.** Beta estimates of the association between ARIP measures and biological age acceleration measure after adjustment for age, sex, race/ethnicity, and CMV status. **Figure S6.** Odds ratios and 95% CI of association of multimorbidity levels with individual T-cell subsets per one SD unit increase in ARIP marker. Adjusted for age, sex, race/ethnicity, CMV status, smoking status, and BMI. The solid black line along OR of 1 indicates no association. **Table S4.** Estimates of association of immunosenescence measures with age-related outcomes. **Table S5.** Associations between self-rated health and ARIP measures after adjustment for age, sex, race/ethnicity, CMV status, smoking status, and BMI.

**Additional file 2.**

## Data Availability

The datasets generated and/or analyzed during the current study are available in the HRS repository at https://hrsdata.isr.umich.edu/data-products/public-survey-data and https://hrsdata.isr.umich.edu/data-products/sensitive-health.
