# Supplementary material for: Evaluation of T-cell aging-related immune phenotypes in the context of biological aging and multimorbidity in the Health and Retirement Study
Source: Immun Ageing. 2022 Jul 20;19:33. doi: 10.1186/s12979-022-00290-z (PMC9297609; doi:10.1186/s12979-022-00290-z)
Supplement: Supplementary file 1 — Additional file 1: Table S1. T cell subset definitions measured in the Health and Retirement Study. Figure S1. Pearson correlation heatmap between individual T-cell subsets measured in the Health and Retirement Study. Figure S2. Association of individual T-cell subsets with chronological age. Age is used as the dependent variable in survey linear regression models. The beta estimates are estimated per one standard deviation unit of individual T-cell subsets. The models are adjusted for sex, race/ethnicity, and CMV status. The solid black line along 0 indicates no association. Figure S3. Scatterplot of ARIP measures with chronological age with a LOWESS curve. Figure S4. Association of individual T-cell subsets with biological age. Biological age is used as the dependent variable in survey linear regression models. The beta estimates are estimated per one standard deviation unit of individual T-cell subsets. The models are adjusted for chronological sex, race/ethnicity, and CMV status. The solid black line along 0 indicates no association. Figure S5. Scatterplot of ARIP measures with biological age with a LOWESS curve. Table S2. Beta estimates of the association between the additional ARIP measures with chronological age and biological age. Table S3. Beta estimates of the association between ARIP measures and biological age acceleration measure after adjustment for age, sex, race/ethnicity, and CMV status. Figure S6. Odds ratios and 95% CI of association of multimorbidity levels with individual T-cell subsets per one SD unit increase in ARIP marker. Adjusted for age, sex, race/ethnicity, CMV status, smoking status, and BMI. The solid black line along OR of 1 indicates no association. Table S4. Estimates of association of immunosenescence measures with age-related outcomes. Table S5. Associations between self-rated health and ARIP measures after adjustment for age, sex, race/ethnicity, CMV status, smoking status, and BMI. [file 12979_2022_290_MOESM1_ESM.docx]

# ­­Evaluation of T-cell aging-related immune phenotypes in the context of biological aging and multimorbidity in the Health and Retirement Study

# **Ramya Ramasubramanian^1^, Helen C.S. Meier^2^, Sithara Vivek^3^, Eric Klopack^4^, Eileen M. Crimmins^4^_,_ Jessica Faul^2^, Janko** Nikolich-Žugich^5^_,_ **Bharat Thyagarajan^3^**

^1^Division of Epidemiology and Community Health, University of Minnesota School of Public Health, Minneapolis, MN, USA

^2^Institute for Social Research, Survey Research Center, University of Michigan, Ann Arbor, MI

^3^Department of Laboratory Medicine and Pathology, University of Minnesota, Minneapolis MN

^4^ Leonard Davis School of Gerontology, University of Southern California, Los Angeles, CA

^5^Department of Immunobiology and the University of Arizona Center on Aging, University of Arizona College of Medicine-Tucson, Tucson, AZ

Corresponding author

Dr. Bharat Thyagarajan

University of Minnesota

Department of Laboratory Medicine and Pathology

E-mail Address: thya0003@umn.edu

Supplementary Table 1: T cell subset definitions measured in the Health and Retirement Study

| **Cell type** | **Surface markers used** | **Parent Population** |
| --- | --- | --- |
| T cells | CD3+ CD19- | Single, Live lymphocytes |
| Cytotoxic T cells | CD3+ CD19- CD8+ CD4- | T cells |
| T_N_ (Naïve) cytotoxic T cells | CD3+ CD19- CD8+ CD4- CD45RA+ CCR7+ CD28+ | Cytotoxic T cells |
| T_EM_ (Effector Memory) cytotoxic T cells | CD3+ CD19- CD8+ CD4- CD45RA- CCR7- CD28- | Cytotoxic T cells |
| T_EMRA_ cytotoxic T cells | CD3+ CD19- CD8+ CD4- CD45RA+ CCR7- CD28- | Cytotoxic T cells |
| T_CM_ (Central Memory) cytotoxic T cells | CD3+ CD19- CD8+ CD4- CD45RA- CCR7+ CD28+ | Cytotoxic T cells |
| Helper T cells | CD3+ CD19- CD4+ CD8- | T cells |
| T_N_ (Naïve) helper T cells | CD3+ CD19- CD4+ CD8- CD45RA+ CCR7+ CD28+ | Helper T cells |
| T_EM_ (Effector Memory) helper T cells | CD3+ CD19- CD4+ CD8- CD45RA- CCR7- CD28- | Helper T cells |
| T_EMRA_ helper T cells | CD3+ CD19- CD4+ CD8- CD45RA+ CCR7- CD28- | Helper T cells |
| T_CM_ (Central Memory) helper T cells | CD3+ CD19- CD4+ CD8- CD45RA- CCR7+ CD28+ | Helper T cells |

Supplementary figure 1: Pearson correlation heatmap between individual T-cell subsets measured in the Health and Retirement Study.


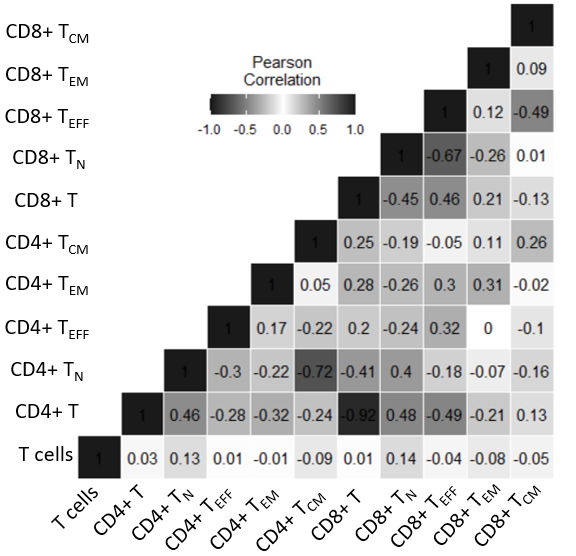


Supplementary figure 2: Association of individual T-cell subsets with chronological age. Age is used as the dependent variable in survey linear regression models. The beta estimates are estimated per one standard deviation unit of individual T-cell subsets. The models are adjusted for sex, race/ethnicity, and CMV status. The solid black line along 0 indicates no association.


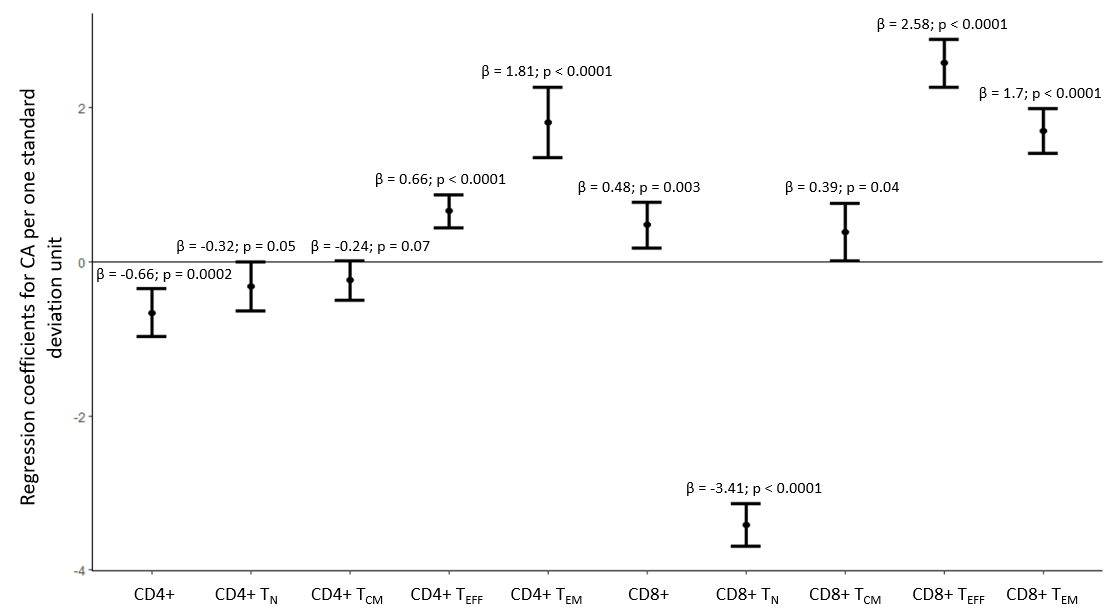


Supplementary Figure 3: Scatterplot of ARIP measures with chronological age with a LOWESS curve.


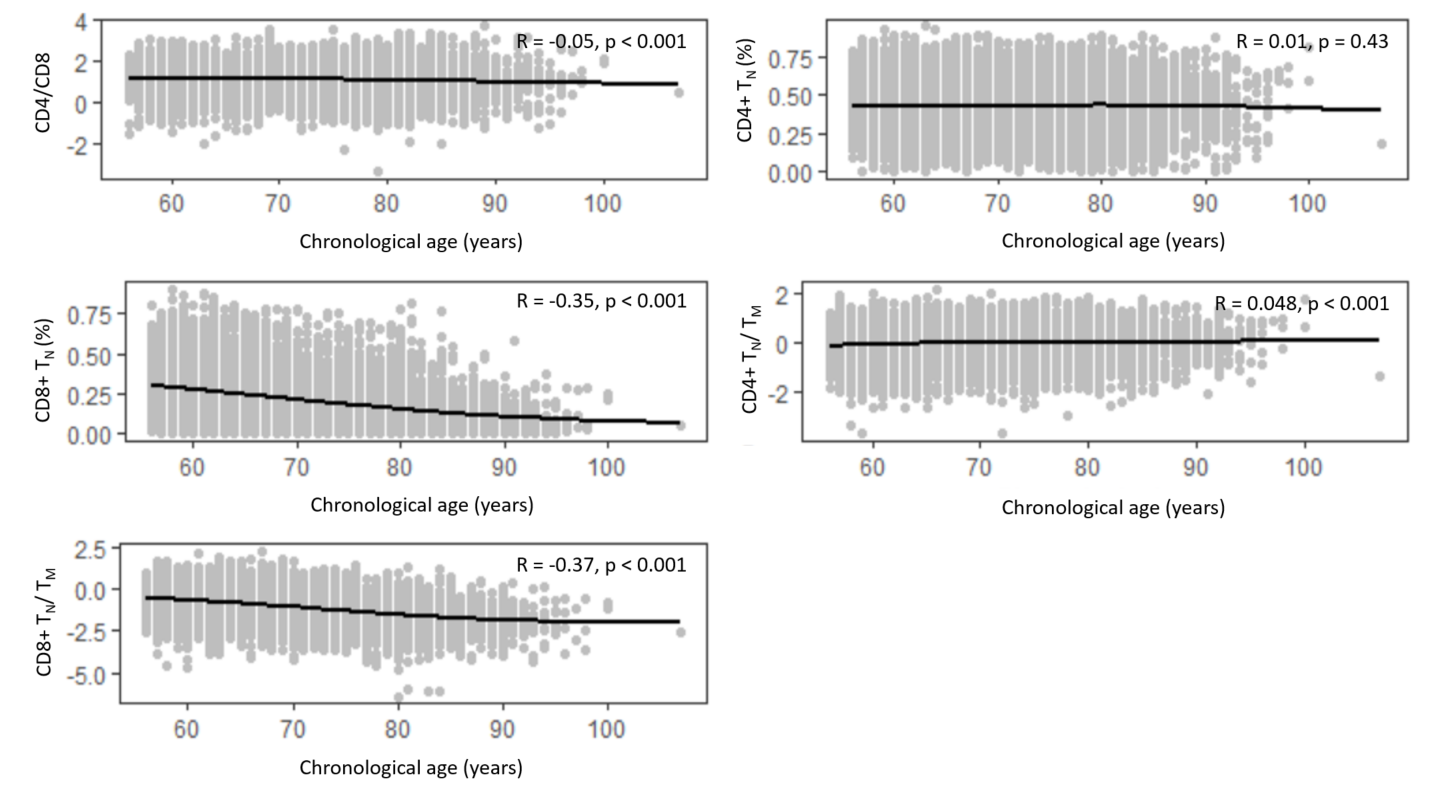


Supplementary figure 4: Association of individual T-cell subsets with biological age. Biological age is used as the dependent variable in survey linear regression models. The beta estimates are estimated per one standard deviation unit of individual T-cell subsets. The models are adjusted for chronological sex, race/ethnicity, and CMV status. The solid black line along 0 indicates no association.


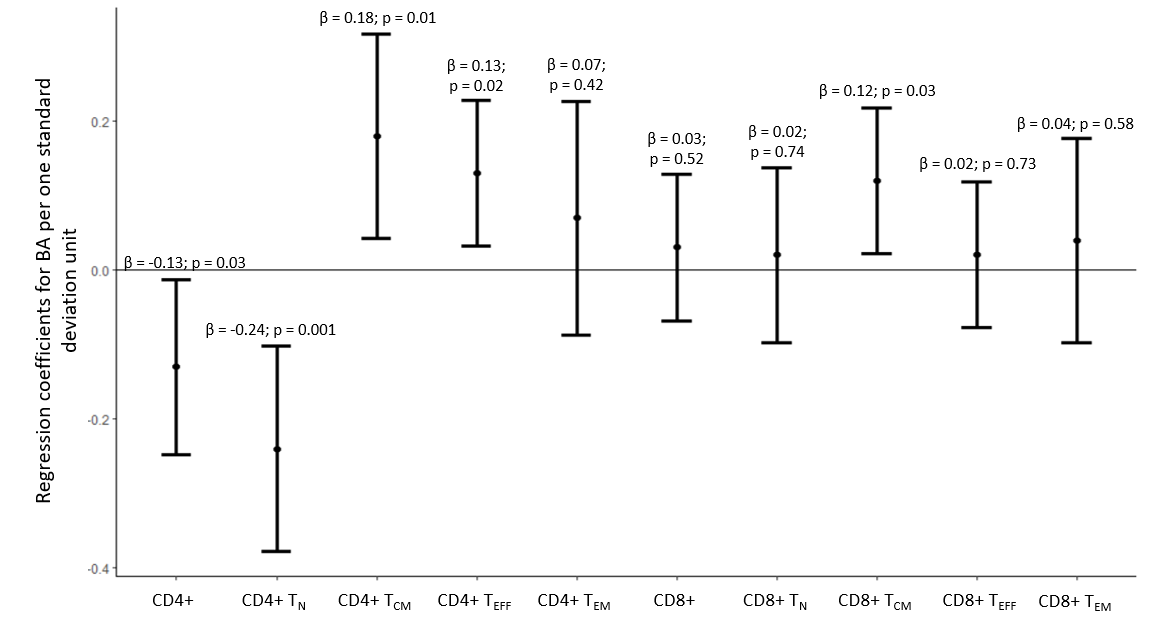


Supplementary Figure 5: Scatterplot of ARIP measures with biological age with a LOWESS curve.


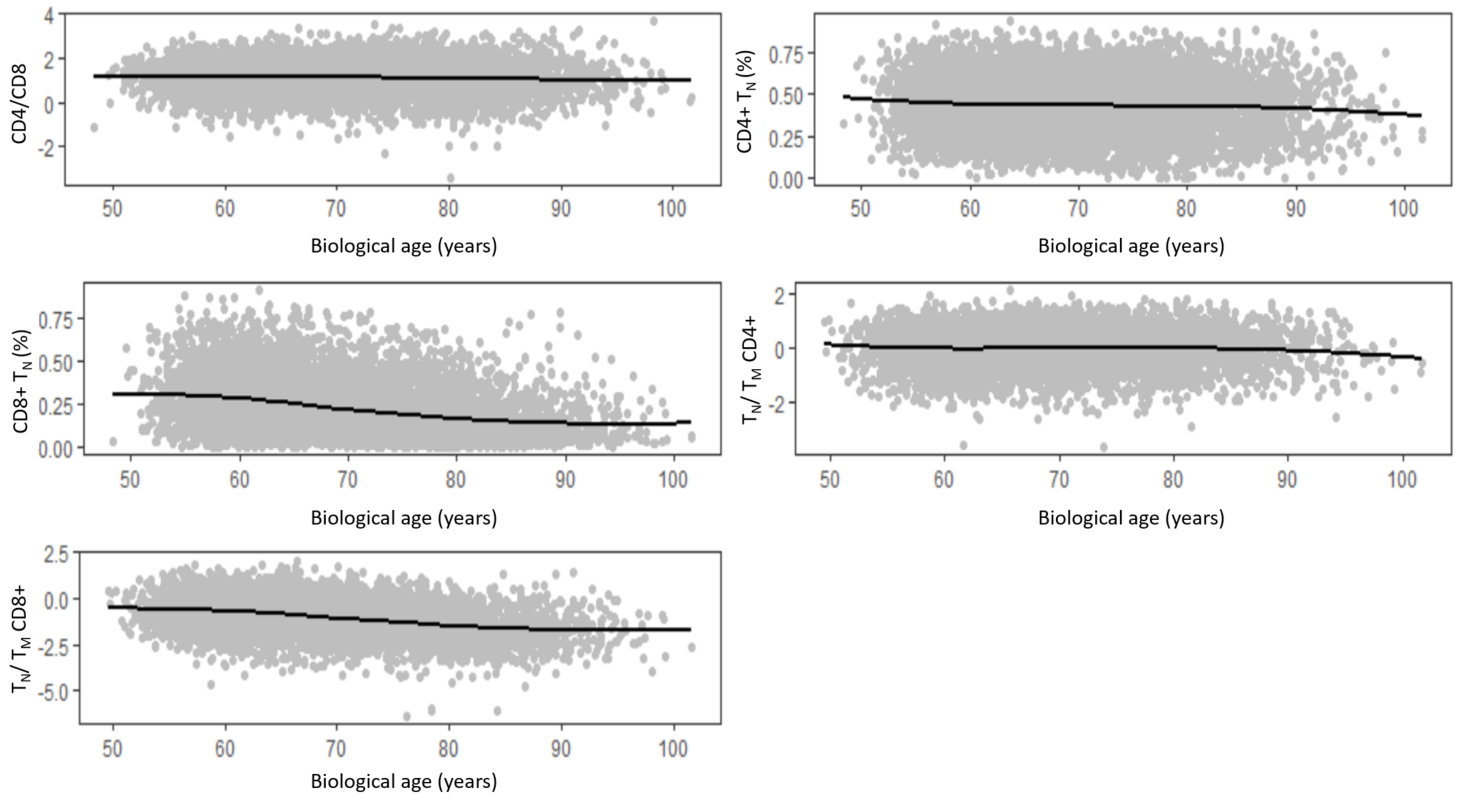


Supplementary table 2 – Beta estimates of the association between the additional ARIP measures with chronological age and biological age.

| **Measure** | **Chronological age (n= 9102)** (68.54 ± 10.23) | **Biological age (n= 7510)**  (69.00 ± 12.35) |
| --- | --- | --- |
| T_N_ CD4+/ T_N_ CD8+ | 2.86 ± 0.16 (<0.0001) | -0.20 ± 0.06 (0.001) |
| T_N_ + T_CM_ CD4+ | -2.31 ± 0.23 (<0.0001) | -0.09 ± 0.06 (0.13) |
| T_N_ + T_CM_ CD8+ | -3.71 ± 0.20 (<0.0001) | 0.11 ± 0.06 (0.06) |

Supplementary Table 3: Beta estimates of the association between ARIP measures and biological age acceleration measure after adjustment for age, sex, race/ethnicity, and CMV status.

| **Measure** | **OR (95% CI) for the older (n=3189) vs younger age (n=4321) acceleration measure** |
| --- | --- |
| CD4/CD8 | 0.97 (95% CI: 0.97 – 1.04; p=0.39) |
| CD4+ T_N_/T_M_ | 0.92 (95% CI: 0.84-1.00; p=0.05) |
| CD8+ T_N_/T_M_ | 1.04 (95% CI: 0.98-1.11; p=0.19) |
| CD8+ T_N_ | 0.99 (95% CI: 0.92 – 1.06; p=0.71) |
| CD4+ T_N_ | 0.89 (95% CI: 0.83 – 0.95; p=0.001) |
| CD4+ T_CM_ | 1.09 (95% CI: 1.02 – 1.17; p=0.01) |

Supplementary figure 6: Odds ratios and 95% CI of association of multimorbidity levels with individual T-cell subsets per one SD unit increase in ARIP marker. Adjusted for age, sex, race/ethnicity, CMV status, smoking status, and BMI. The solid black line along OR of 1 indicates no association.


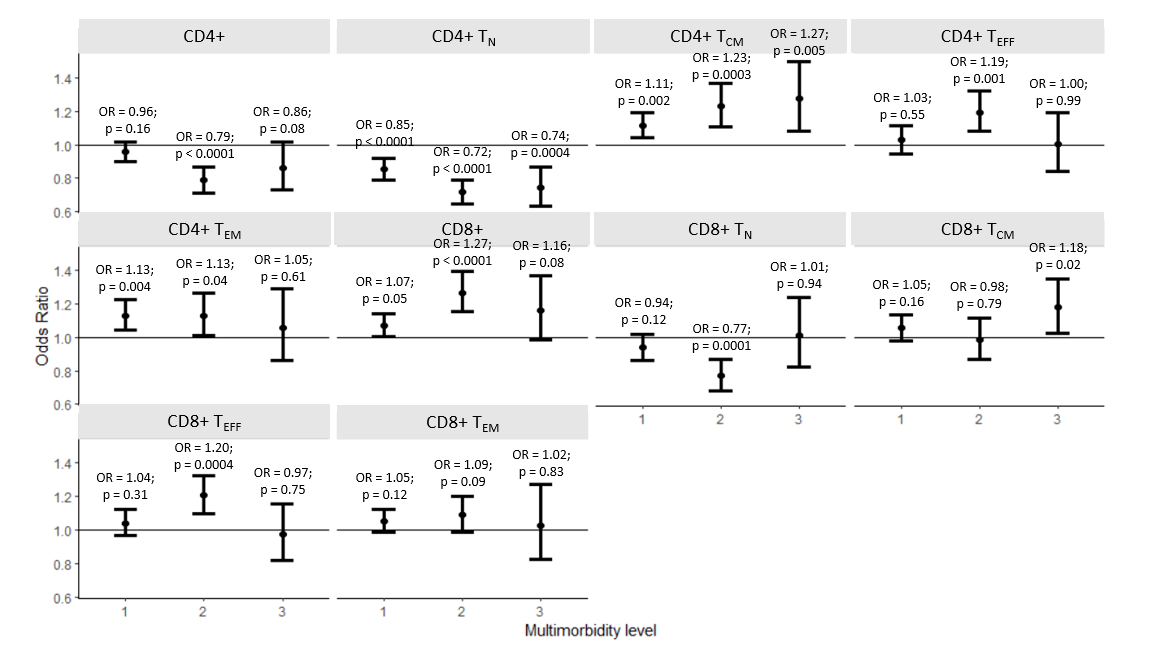


Supplementary table 4: Estimates of association of immunosenescence measures with age-related outcomes

| ARIP measures |  | | |
| --- | --- | --- | --- |
|  | **Multimorbidity – 2016** | | |
|  | Model 1^a^ | Model 2^b^ | Model 3^c^ |
|  | OR (95% CI) | OR (95% CI) | OR (95% CI) |
| CD4/CD8 ratio | 0.80 (0.75 – 0.84) | 0.84 (0.79-0.90) | 0.83 (0.77 – 0.90) |
| CD4+ T_N_ / CD8+ T_N_ | 0.97 (0.91 – 1.03) | 0.87 (0.81-0.93) | 0.88 (0.81-0.96) |
| CD4+ T_N_ + T_CM_ | 0.80 (0.75 – 0.85) | 0.90 (0.85 – 0.96) | 0.85 (0.79-0.91) |
| CD8+ T_N_ + T_CM_ | 0.88 (0.82 – 0.95) | 1.04 (0.96 – 1.13) | 1.01 (0.91 – 1.12) |
| CD4+ T_N_/T_M_ | 0.72 (0.64 – 0.80) | 0.71 (0.64 - 0.80) | 0.78 (0.68 – 0.89) |
| CD8+ T_N_/T_M_ | 0.72 (0.67-0.79) | 0.82 (0.76-0.89) | 0.82 (0.75-0.89) |
| CD8+ T_N_ | 0.79 (0.75 – 0.83) | 0.93 (0.88 – 1.00) | 0.85 (0.77 – 0.93) |
| CD4+ T_N_ | 0.74 (0.68-0.79) | 0.75 (0.70 – 0.81) | 0.78 (0.71 – 0.85) |
| CD4+ T_CM_ | 1.22 (1.13 – 1.31) | 1.23 (1.14 – 1.34) | 1.18 (1.07 – 1.30) |
| **Associations of immunosenescence measures with individual components of multimorbidity in 2016** | | | |
|  | **Type II diabetes – 2016** | | |
|  | Model 1^a^ | Model 2^b^ | Model 3^c^ |
|  | OR (95% CI) | OR (95% CI) | OR (95% CI) |
| CD4/CD8 ratio | 0.90 (0.85 – 0.96) | 0.97 (0.91 – 1.03) | 0.96 (0.88 – 1.05) |
| CD4+ T_N_ / CD8+ T_N_ | 0.92 (0.86 – 0.98) | 0.93 (0.86 – 1.00) | 0.92 (0.84 – 1.01) |
| CD4+ T_N_ + T_CM_ | 1.02 (0.96 – 1.08) | 1.10 (1.04 – 1.16) | 1.05 (0.98-1.12) |
| CD8+ T_N_ + T_CM_ | 1.03 (0.97 – 1.10) | 1.08 (1.00 - 1.16) | 1.09 (0.99 -1.20) |
| CD4+ T_N_/T_M_ | 0.69 (0.64 – 0.74) | 0.73 (0.68 – 0.79) | 0.82 (0.75 – 0.90) |
| CD8+ T_N_/T_M_ | 0.83 (0.78 – 0.89) | 0.87 (0.80 – 0.94) | 0.90 (0.82 – 0.99) |
| CD8+ T_N_ | 0.94 (0.88 – 1.00) | 0.99 (0.92 – 1.07) | 0.86 (0.79-0.93) |
| CD4+ T_N_ | 0.75 (0.69 – 0.81) | 0.80 (0.74 – 0.86) | 0.82 (0.76-0.90) |
| CD4+ T_CM_ | 1.33 (1.25 – 1.41) | 1.27 (1.20 – 1.35) | 1.20 (1.12 – 1.29) |
|  | **Heart Disease - 2016** | | |
|  | Model 1^a^ | Model 2^b^ | Model 3^c^ |
|  | OR (95% CI) | OR (95% CI) | OR (95% CI) |
| CD4/CD8 ratio | 0.92 (0.88 – 0.96) | 0.95 (0.90-1.00) | 0.93 (0.87 – 0.99) |
| CD4+ T_N_ / CD8+ T_N_ | 1.13 (1.07 – 1.20) | 0.98 (0.93 – 1.03) | 0.99 (0.93 – 1.05) |
| CD4+ T_N_ + T_CM_ | 0.83 (0.78 – 0.88) | 0.95 (0.90 1.00) | 0.92 (0.87-0.98) |
| CD8+ T_N_ + T_CM_ | 0.81 (0.76 – 0.87) | 0.99 (0.92-1.06) | 0.98 (0.90 – 1.06) |
| CD4+ T_N_/T_M_ | 0.90 (0.83 – 0.98) | 0.89 (0.81 – 0.98) | 0.92 (0.84 – 1.02) |
| CD8+ T_N_/T_M_ | 0.78 (0.73 – 0.83) | 0.90 (0.84 – 0.97) | 0.91 (0.83-0.98) |
| CD8+ T_N_ | 0.76 (0.72 – 0.81) | 0.94 (0.89 – 0.99) | 0.94 (0.86 – 1.03) |
| CD4+ T_N_ | 0.88 (0.83 – 0.93) | 0.90 (0.84 – 0.95) | 0.92 (0.86 – 0.97) |
| CD4+ T_CM_ | 1.08 (1.02 – 1.14) | 1.10 (1.04 – 1.16) | 1.06 (1.00 -1.12) |
|  | **Lung Disease - 2016** | | |
|  | Model 1^a^ | Model 2^b^ | Model 3^c^ |
|  | OR (95% CI) | OR (95% CI) | OR (95% CI) |
| CD4/CD8 ratio | 0.93 (0.85 – 1.01) | 0.97 (0.89 – 1.05) | 0.97 (0.88 – 1.07) |
| CD4+ T_N_ / CD8+ T_N_ | 0.94 (0.87 – 1.03) | 0.91 (0.83 – 0.99) | 0.94 (0.85 – 1.04) |
| CD4+ T_N_ + T_CM_ | 0.93 (0.86 – 1.01) | 0.95 (0.87 – 1.03) | 0.88 (0.80 – 0.97) |
| CD8+ T_N_ + T_CM_ | 1.01 (0.93 – 1.11) | 1.05 (0.94 – 1.16) | 0.92 (0.81 – 1.05) |
| CD4+ T_N_/T_M_ | 0.83 (0.74 – 0.94) | 0.81 (0.71 – 0.92) | 0.88 (0.76-1.03) |
| CD8+ T_N_/T_M_ | 0.92 (0.84 – 1.02) | 0.97 (0.88 – 1.07) | 0.95 (0.84 – 1.07) |
| CD8+ T_N_ | 0.94 (0.87 – 1.02) | 0.97 (0.88 – 1.06) | 0.94 (0.83 – 1.08) |
| CD4+ T_N_ | 0.84 (0.76 – 0.93) | 0.83 (0.75 – 0.93) | 0.90 (0.79 – 1.01) |
| CD4+ T_CM_ | 1.12 (0.99 – 1.26) | 1.16 (1.02 – 1.32) | 1.09 (0.94 – 1.28) |
|  | **Stroke - 2016** | | |
|  | Model 1^a^ | Model 2^b^ | Model 3^c^ |
|  | OR (95% CI) | OR (95% CI) | OR (95% CI) |
| CD4/CD8 ratio | 0.90 (0.81 – 1.00) | 1.00 (0.89 – 1.12) | 0.97 (0.85-1.10) |
| CD4+ T_N_ / CD8+ T_N_ | 1.03 (0.92 – 1.15) | 0.97 (0.86 – 1.10) | 0.98 (0.87 – 1.10) |
| CD4+ T_N_ + T_CM_ | 0.86 (0.79 – 0.93) | 0.98 (0.89 – 1.09) | 0.95 (0.86 – 1.06) |
| CD8+ T_N_ + T_CM_ | 0.86 (0.77 – 0.96) | 0.99 (0.86 – 1.12) | 0.99 (0.87 – 1.14) |
| CD4+ T_N_/T_M_ | 0.83 (0.73 – 0.95) | 0.89 (0.77 – 1.02) | 0.92 (0.80 – 1.06) |
| CD8+ T_N_/T_M_ | 0.87 (0.79 – 0.95) | 1.04 (0.91 – 1.18) | 1.04 (0.89 – 1.20) |
| CD8+ T_N_ | 0.83 (0.77 – 0.89) | 0.96 (0.86 – 1.06) | 1.03 (0.87 – 1.21) |
| CD4+ T_N_ | 0.83 (0.74 – 0.93) | 0.93 (0.82 – 1.05) | 0.96 (0.85 – 1.08) |
| CD4+ T_CM_ | 1.17 (1.03 – 1.32) | 1.12 (0.99 – 1.27) | 1.09 (0.96 – 1.24) |
|  | **Cancer - 2016** | | |
|  | Model 1^a^ | Model 2^b^ | Model 3^c^ |
|  | OR (95% CI) | OR (95% CI) | OR (95% CI) |
| CD4/CD8 ratio | 0.83 (0.77 – 0.90) | 0.81 (0.75 – 0.88) | 0.79 (0.72-0.86) |
| CD4+ T_N_ / CD8+ T_N_ | 0.94 (0.86 – 1.04) | 0.78 (0.71 – 0.86) | 0.79 (0.72 – 0.87) |
| CD4+ T_N_ + T_CM_ | 0.65 (0.60 – 0.71) | 0.69 (0.63 – 0.76) | 0.66 (0.61 – 0.72) |
| CD8+ T_N_ + T_CM_ | 0.79 (0.71 – 0.87) | 0.92 (0.83 – 1.02) | 0.89 (0.80 – 1.00) |
| CD4+ T_N_/T_M_ | 0.87 (0.79 – 0.95) | 0.81 (0.74 – 0.89) | 0.81 (0.74 – 0.89) |
| CD8+ T_N_/T_M_ | 0.82 (0.76 – 0.89) | 0.92 (0.86 – 1.00) | 0.89 (0.82 – 0.96) |
| CD8+ T_N_ | 0.75 (0.70 – 0.82) | 0.92 (0.84 – 1.00) | 0.92 (0.84 – 1.01) |
| CD4+ T_N_ | 0.80 (0.73 – 0.87) | 0.76 (0.70 – 0.83) | 0.75 (0.68 – 0.82) |
| CD4+ T_CM_ | 1.07 (0.98 – 1.17) | 1.12 (1.03 – 1.22) | 1.12 (1.03 – 1.23) |
|  | **Incident multimorbidity – 2018 and 2020** | | |
|  | Model 1^a^ | Model 2^b^ | Model 3^c^ |
|  | Hazard Ratio (95% CI) | Hazard Ratio (95% CI) | Hazard Ratio (95% CI) |
| CD4/CD8 ratio | 0.93 (0.85 – 1.02) | 0.95 (0.86 – 1.05) | 0.94 (0.85 – 1.04) |
| CD4+ T_N_ / CD8+ T_N_ | 0.98 (0.90 – 1.08) | 0.95 (0.87 – 1.05) | 0.96 (0.87 – 1.06) |
| CD4+ T_N_ + T_CM_ | 0.99 (0.92 – 1.06) | 1.07 (0.98 – 1.16) | 1.04 (0.95 – 1.13) |
| CD8+ T_N_ + T_CM_ | 0.99 (0.90 – 1.11) | 1.09 (0.98 – 1.22) | 1.07 (0.95 – 1.19) |
| CD4+ T_N_/T_M_ | 0.82 (0.75 – 0.90) | 0.86 (0.77 – 0.95) | 0.91 (0.82 – 1.02) |
| CD8+ T_N_/T_M_ | 0.87 (0.82 – 0.92) | 0.93 (0.87 – 1.00) | 0.94 (0.87 – 1.02) |
| CD8+ T_N_ | 0.92 (0.84 – 1.01) | 0.99 (0.89 – 1.09) | 0.99 (0.89 – 1.10) |
| CD4+ T_N_ | 0.87 (0.80 – 0.95) | 0.90 (0.82 – 0.99) | 0.91 (0.82 – 1.01) |
| CD4+ T_CM_ | 1.17 (1.09 – 1.26) | 1.14 (1.06 – 1.23) | 1.09 (1.01 – 1.18) |
| ^a^Model 1 assessed bivariate associations.  ^b^Model 2 adjusted for age, sex, race, and CMV status.  ^c^Model 3 additionally adjusted for smoking status, BMI.  Note: Effect estimates are per one SD unit increase in immunosenescence measures. | | | |

Supplementary Table 5: Associations between self-rated health and ARIP measures after adjustment for age, sex, race/ethnicity, CMV status, smoking status, and BMI.

| **Measure** | **Odds ratio (bad (n=2486) vs good self-rated health (6108))** |
| --- | --- |
| CD4/CD8 | 0.89 (95% CI: 0.82 – 0.96); p=0.002 |
| T_N_/T_M_ CD4+ | 0.84 (95% CI: 0.78 – 0.91); p <0.0001 |
| T_N_/T_M_ CD8+ | 0.88 (95% CI: 0.80 – 0.96); p=0.01 |
| T_N_ CD8+ | 0.91 (95% CI: 0.83 – 1.01); p=0.07 |
| T_N_ CD4+ | 0.83 (95% CI: 0.77 – 0.89); p<0.0001 |
| T_CM_ CD4+ | 1.12 (95% CI: 1.04 – 1.20); p =0.004 |
